# Efficient and Secure Key Distribution Protocol for Wireless Sensor Networks

**DOI:** 10.3390/s18103569

**Published:** 2018-10-21

**Authors:** Majid R. Alshammari, Khaled M. Elleithy

**Affiliations:** Department of Computer Science and Engineering, University of Bridgeport, 126 Park Ave, Bridgeport, CT 06604, USA; elleithy@bridgeport.edu

**Keywords:** key distribution, wireless sensor networks, resource-constrained nodes

## Abstract

Modern wireless sensor networks have adopted the IEEE 802.15.4 standard. This standard defines the first two layers, the physical and medium access control layers; determines the radio wave used for communication; and defines the 128-bit advanced encryption standard (AES-128) for encrypting and validating the transmitted data. However, the standard does not specify how to manage, store, or distribute the encryption keys. Many solutions have been proposed to address this problem, but the majority are impractical in resource-constrained devices such as wireless sensor nodes or cause degradation of other metrics. Therefore, we propose an efficient and secure key distribution protocol that is simple, practical, and feasible to implement on resource-constrained wireless sensor nodes. We conduct simulations and hardware implementations to analyze our work and compare it to existing solutions based on different metrics such as energy consumption, storage overhead, key connectivity, replay attack, man-in-the-middle attack, and resiliency to node capture attack. Our findings show that the proposed protocol is secure and more efficient than other solutions.

## 1. Introduction

A wireless sensor network (WSN) is a network composed of resource-constrained devices with the ability to perform sensing and wireless communications, which are called wireless sensor nodes. Due to the low cost and flexibility of WSNs, they are employed in a variety of applications such as agriculture, the environment, health, home and commercial automation, the military and transportation [1,2,3,4,5,6,7,8,9,10,11] and have recently become a promising technology for Internet of Things (IoT) applications [12,13,14,15]. Modern WSNs adopt the IEEE 802.15.4 standard, which specifies the physical layer and the medium access control (MAC) layer for low-rate wireless personal area networks (LR-WPANs). The standarad also determines the radio frequency used for communication and provides four security services: access control, confidentially, integrity and replay protection. The MAC layer handles security for the IEEE 802.15.4 standard and defines the 128-bit advanced encryption standard (AES-128) for encrypting and validating transmitted data. Unfortunately, the standard does not specify how to manage, store, or distribute encryption keys [16]. Many solutions have been proposed to address this problem, but the majority are impractical in resource-constrained devices such as wireless sensor nodes or cause degradation of other metrics.

In this work, we propose an efficient and secure key distribution protocol for WSNs. We utilized the existing cryptographic primitives to design a protocol that is simple, practical and feasible to implement on resource-constrained devices such as wireless sensor nodes. This work extends our preliminary work introduced in [17] by improving its efficiency and security. The contributions of our work can be summarized as follows.

- We introduce a comprehensive classification for the main key distribution and key establishment schemes in WSNs. We classify the schemes into traditional key distribution schemes, including private-key-based schemes and public-key-based schemes, and quantum-based key distribution schemes, including those based on entanglement swapping and teleportation.
- We propose an efficient and secure key distribution protocol that is simple, practical and feasible to implement on resource-constrained devices such as wireless sensor nodes. Because data communication is responsible for most of a node’s energy consumption [18], the proposed protocol utilizes the existing cryptographic primitives and leverages asymmetric encryption to achieve key distribution and node authentication in one step and using only one frame to avoid communication overhead. Moreover, the implementation of the proposed protocol adopts the following techniques: a fast modular exponentiation algorithm (described in Appendix A.4) and a short public exponent. These techniques speed up the node’s data computation, resulting in lower energy consumption.
- We analyze and compare the proposed protocol against different types of schemes using various metrics, including energy consumption, key connectivity, storage overhead, man-in-the-middle attack, replay attack and resiliency to node capture attack. Our methodology (described in Appendix A.1) combines simulations, hardware implementations and practical models to calculate both the energy consumption of sensor nodes and the energy consumption caused by wireless channel effects.
- We visualize and analyze the key connectivity and the impact of node capture attack using a graph. We model a WSN as a graph and then implement the proposed protocol and the corresponding schemes on the graph to investigate their key connectivity and the impact of node capture attack on the key connectivity.
- We conduct a formal verification using an automatic cryptographic protocol verifier, ProVerif. We utilize ProVerif to prove the security and soundness of the proposed protocol in formal models. We verify the reachability and secrecy, correspondence assertions (authentication) and observational equivalences.

The remainder of this paper is organized as follows: The next section introduces a classification for WSN key distribution schemes and work related to the proposed protocol. Section 3 describes the proposed protocol. Section 4 presents our findings and analyses. Section 5 describes the formal verification of our proposed protocol, and Section 6 concludes the paper.

## 2. Related Work

**Figure 1:**
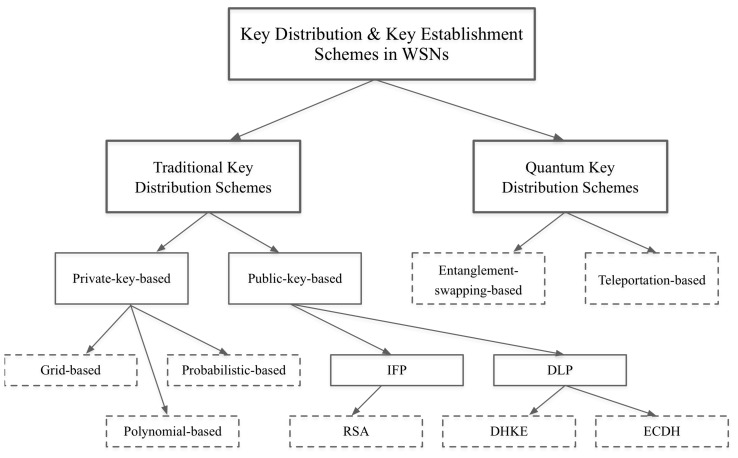
Classification of key distribution schemes in WSNs.

Key distribution schemes in WSNs have been comprehensively studied in the literature. The authors of [19,20,21,22] provided detailed surveys. However, in this study, we present a comprehensive overview of the existing key distribution and key establishment schemes in WSNs, which we classify into two domains. The first domain includes traditional key-based distribution schemes, which can be further classified into private-key-based and public-key-based schemes. Private-key-based schemes can be subcategorized into grid-based, polynomial-based and probabilistic schemes. Public-key-based key distribution schemes can be subcategorized based on an integer factorization problem (IFP) or on a discrete logarithm problem (DLP). The second domain includes quantum-based key distribution schemes, which can be further classified into entanglement-swapping-based and teleportation-based schemes. Figure 1 depicts this classification hierarchy, and Table 1 defines our evaluation metrics.

Grid-based schemes address a WSN of size *n* as an $\sqrt{n}\cdot \sqrt{n}$ matrix or grid. In this subcategory, each node in the WSN is assigned to a unique intersection (i,j) in the grid [23,24,25]. An early example was called Peer Intermediaries for Key Establishment in Sensor Networks (PIKE) [26]. PIKE represents a sensor network of size *n* by an $\sqrt{n}\cdot \sqrt{n}$ matrix and uses some sensor nodes as trusted intermediaries for key distribution. Each sensor has an ID in the form of (x,y) based on its position in the matrix. Moreover, each node is loaded with a pairwise secret key shared only with each node in the two sets:

(1) $$\left(i,y\right)\;\forall \;i\in \left\{1,2,3,\ldots ,\sqrt{n-1}\right\}$$

(2) $$\left(x,j\right)\;\forall \;i\in \left\{1,2,3,\ldots ,\sqrt{n-1}\right\}.$$

Keys are deployed such that in any pair *A* and *B*, at least one node *C* exists that shares a pairwise key with both *A* and *B*. However, this approach suffers from key dependency because one inoperable or missing node would impact the network connectivity. Additionally, the search process for intermediary nodes consumes a large amount of energy because it involves sending many frames to other sensor nodes searching for a node that shares a pairwise key. Moreover, this approach requires each sensor node to store $2\left(\sqrt{n-1}\right)$ keys.

Polynomial-based schemes depend on storing polynomials on wireless sensor nodes that are used for key generation. In [27], this process was described as a threshold scheme (R,K). The threshold is a shared security scheme that divides a message into *K* parts, where *R* is the minimum number of parts required to reconstruct the original message. The author of [28], proposed a sharing security scheme that used a polynomial equation in a finite field to construct a threshold scheme. In this scheme, an arbitrary polynomial of degree R−1 was generated in the following form:

(3) $$\left(ax^{R-1}+bx^{(R-1)-1}+\ldots +m\right)mod\;p,$$

where *p* is a public prime number that is greater than the coefficients, *m* is the message, and *a* and *b* are randomly chosen coefficients. Many other forms of polynomial-based key distribution schemes have been proposed [29,30,31,32,33,34]. In [35], the authors proposed a polynomial-based key management scheme consisting of three phases. In the first phase, sensor nodes discover the neighboring nodes and elect a cluster head (CH). In the second phase, when a sensor node wants to acquire a secure communication channel through the base station (BS), it sends a registration request. The third phase generates a triple key. Subsequently, when a sensor node wants to communicate with the CH and BS, the key is calculated based on a given polynomial and coefficients in a finite group. In this approach, sensor nodes consume large amounts of energy during the discovery and cluster-electing phases. Additionally, key connectivity is affected by node capture attacks.

Probabilistic-based schemes rely on the probability of two sensor nodes sharing a common key to establish a communication link. In [36], the authors proposed a probabilistic key-based management protocol. This scheme consists of three phases: key predistribution, shared-key discovery and path-key establishment. During the key predistribution phase, a large pool of keys *p* and their identifiers are generated. Then, a random set of keys *k* and their identifiers are drawn out of the key pool *p* to form a key ring for each sensor node based on the following formula:

(4) $$P_{key}=1-\left(\frac{{\left(\left(P-k\right)!\right)}^{2}}{\left((P-2k)!P!\right)}\right),$$

where $P_{key}$ is the probability of two nodes sharing a common key. Next, the key rings are loaded into each of the sensor node memories, and the key identifiers are saved on a trusted controller node along with the associated sensor identifiers. In the shared-key discovery phase, two sensor nodes can discover a shared key by broadcasting a list of their key rings. Additionally, the two sensor nodes can hide the key sharing patterns by broadcasting a list, li={α||Eki(α)||i=1,…,k}, for every key on the key ring, where α is a challenge. The ability of the receiver to decrypt Eki(α) will reveal the challenge α and then establish a shared key with the sender. In the path-key establishment phase, a path key is assigned to each pair of sensor nodes that do not share a key but are connected to other sensor nodes at the end of the shared-key discovery phase. However, this approach consumes large amounts of energy because finding a common key between two sensor nodes requires broadcasting many frames. Additionally, storing the key ring requires large amounts of memory, especially when the probability of sensor nodes sharing a common key is certain.

Public-key-based schemes are another class of the traditional key distribution schemes in WSNs. Practical public-key-based schemes depend on two major families of problems: IFPs such as the Rivest–Shamir–Adleman (RSA) cryptosystem and DLPs such as Diffie-Hellman key exchange (DHKE) and elliptic curve Diffie-Hellman (ECDH). The authors of [37] discussed using public infrastructure such as RSA to improve the security of WSNs. The study considered the WSN topology as a set of wirelessly connected sensor nodes that report collected data to the base station. However, wireless sensor nodes are resource-constrained devices, and a straightforward implementation of IFP or DLP without appropriate modifications is energy and memory intensive. In [38], the authors proposed a public-key-based key distribution scheme using ECDH. The scheme consists of two phases: a predeployment phase and a postdeployment phase. In the predeployment phase, sensor nodes are configured with elliptic curve (EC) parameters and the basepoint *G*. Then, $\alpha_{n}$ is generated to calculate $P_{n}=\alpha_{n}G$ for all *n* nodes. Next, $\alpha_{n}$ is stored in each corresponding node, and all $P_{n}$ are stored in the sink node. In the postdeployment phase, the sink creates a new secret key *b* and calculates its public key Q=bG. The public key is then broadcast to all sensor nodes. Each sensor node calculates a new key $k_{n}=\alpha_{n}Q$, whereas the sink calculates a new key $k_{n}=bP_{n}$. The downside of this approach is that each node must store all the EC parameters such as the field over which the curve is defined, the α and *b* values that defined the curve and the generator point *G*.

Quantum-based schemes rely on the laws of physics and require special hardware. Nevertheless, many authors have proposed solutions. For example, entanglement swapping was adopted in [39]. This scheme utilizes a third party, called a base station, to perform entanglement swapping among sensor nodes. Each sensor node shares *n* qubits with the base station, and the base station shares *m* qubits with each sensor node. When two sensor nodes *x* and *y* are separately entangled with the base station, the base station performs entanglement swapping, allowing sensor node *x* and sensor node *y* to become entangled. As another example, the authors of [40] proposed a scheme that includes Einstein-Podolsky-Rosen (EPR)-pair distribution and quantum authentication. This scheme allows a sensor node to teleport quantum information to any other sensor node in the network. The entanglement-swapping-based and teleportation-based schemes rely on quantum cryptography, which has been proven in prior literature to be secure unless the laws of physics have been defeated. However, these schemes require entangled qubits to function, and applying entangled qubits in resource-constrained devices such as WSNs is not yet practical with existing technology.

## 3. Proposed Protocol

The proposed protocol includes four phases: *a pre–deployment phase*, *a key distribution phase*, *a post-key distribution phase*, and *a key refreshment phase*. Table 2 presents the notations used to describe the proposed protocol.

### 3.1. Pre-Deployment Phase


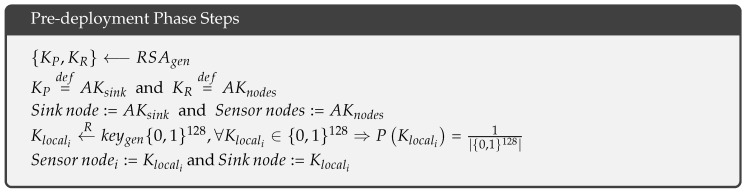


The pre-deployment phase of the protocol consists of five offline steps. The first step is the generation of an asymmetric key pair $\left\{K_{P},K_{R}\right\}⟵RSA_{gen}$, where $RSA_{gen}$ is the RSA key generation algorithm. In the second step, $K_{P}$ is defined as a sink node key, $AK_{sink}$, and $K_{R}$ is defined as the key for the sensor nodes, $AK_{nodes}$. In the third step, $AK_{sink}$ is loaded into the sink node, and $AK_{nodes}$ is loaded into the sensor nodes. The fourth step involves the generation of a random local key for each sensor node as follows: $K_{local_{i}}\overset{R}{\leftarrow }key_{gen}{\left\{0,1\right\}}^{128}$, where $key_{gen}{\left\{0,1\right\}}^{128}$ is a random key generation algorithm with a key space of ${\left\{0,1\right\}}^{128}$; the key space is a uniform distribution such that $\forall K_{local_{i}}\in {\left\{0,1\right\}}^{128}$, and the probability of each key is $P\left(K_{local_{i}}\right)=\frac{1}{{|\left\{0,1\right\}}^{128}|}$. In the fifth step, $K_{local_{i}}$ is loaded into the corresponding sensor node_i_ and into the sink nodes.

### 3.2. Key Distribution Phase


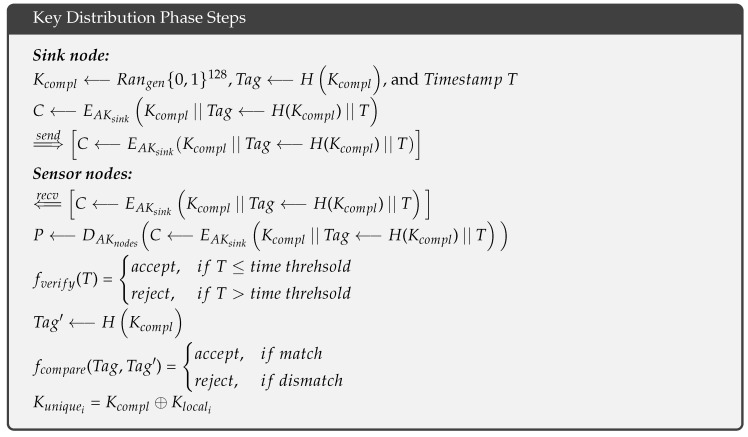


After deploying the sensor nodes, the sink node generates a random complementary key $K_{compl}⟵Ran_{gen}{\left\{0,1\right\}}^{128}$, computes its hash value $Tag⟵H(K_{compl})$, and calculates a timestamp *T*. The sink node then encrypts these values using its asymmetric key $AK_{sink}$. After this task is complete, the sink node sends the cipher to neighboring sensor nodes as follows: $\overset{send}{\Rightarrow }\left[C⟵E_{AK_{sink}}\left(K_{compl}\;||\;Tag⟵H\left(K_{compl}\right)\;\left|\right|\;T\right)\right]$. These neighbors forward the cipher to their neighbors in a multihop fashion until all of the sensor nodes have received the cipher $\overset{recv}{⇐}\left[C⟵E_{AK_{sink}}\left(K_{compl}\;||\;Tag⟵H\left(K_{compl}\right)\;\left|\right|\;T\right)\right]$. Because each sensor node is loaded with the asymmetric key $AK_{nodes}$ in the pre-deployment phase, a sensor node_i_ can decrypt the cipher as follows: $P⟵D_{AK_{nodes}}\left(C⟵E_{AK_{sink}}\left(K_{compl}\;||\;Tag⟵H\left(K_{compl}\right)\;\left|\right|\;T\right)\right)$ and verifies the timestamp $f_{verify}\left(T\right)$ based on a predefined threshold. If the timestamp exceeds the threshold, the sensor node_i_ rejects the cipher. Otherwise, the sensor node_i_ hashes the complementary key ${Tag}^{\prime }⟵H\left(K_{compl}\right)$ and compares it with the received hash $f_{compare}\left(Tag,{Tag}^{\prime }\right)$ to ensure it has not received a modified complementary key $K_{compl}$. If a mismatch is found, the sensor node_i_ rejects the cipher; otherwise, the sensor node_i_ produces its unique key by XORing the complementary key and its local key as follows: $K_{unique_{i}}=K_{compl}⊕K_{local_{i}}$.

### 3.3. Post-Key Distribution Phase


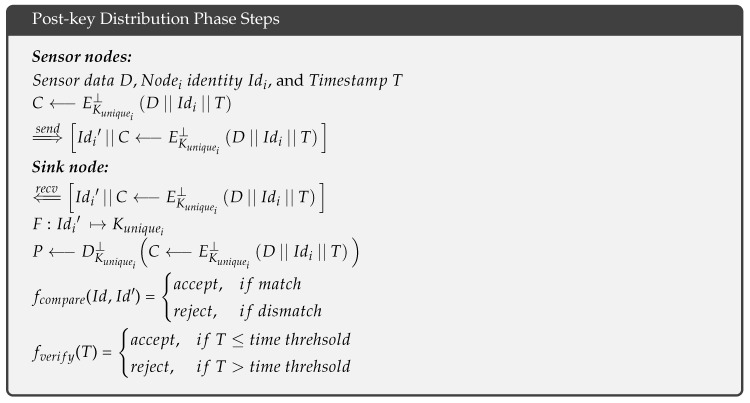


After establishing the key distribution phase, the sensor nodes have already produced their unique keys. Therefore, when sensor node_i_ wants to transmit data *D* to the sink node, it uses its unique key $K_{unique_{i}}$ to encrypt the data *D*, its identity $Id_{i}$, and a timestamp *T*; then, it concatenates the cipher with another copy of its identity $Id_{i}{}^{\prime }$ and sends both to the sink node. This process is described as follows: $\overset{send}{\Rightarrow }\left[Id_{i}{}^{\prime }\;\left|\right|\;C⟵E_{K_{unique_{i}}}^{⊥}\left(D\;||\;Id_{i}\;\left|\right|\;T\right)\right]$, where ⊥ is a probabilistic encryption algorithm. Because this study is concerned with key distribution, the sensor node_i_ can use any secure probabilistic encryption algorithm. When the sink node receives the following cipher $\overset{recv}{⇐}\left[Id_{i}{}^{\prime }\;\left|\right|\;C⟵E_{K_{unique_{i}}}^{⊥}\left(D\;||\;Id_{i}\;\left|\right|\;T\right)\right]$, it uses the concatenated node $Id_{i}{}^{\prime }$ to find the corresponding $K_{unique_{i}}$ as follows: $F:Id_{i}{}^{\prime }\;\mapsto K_{unique_{i}}$. Then, the sink node decrypts the cipher and compares the identities $f_{compare}\left(Id,Id{}^{\prime }\right)$, to ensure that the appropriate $K_{unique_{i}}$ is used and that the cipher is received from an authorized node. If a match is found, the sink node verifies the timestamp *T*, $f_{verify}\left(T\right)$, based on a predefined threshold. When *T* is less than or equal to the threshold, the sink node accepts the sensor data *D*; otherwise, it rejects the sensor data *D*.

### 3.4. Key Refreshment Phase


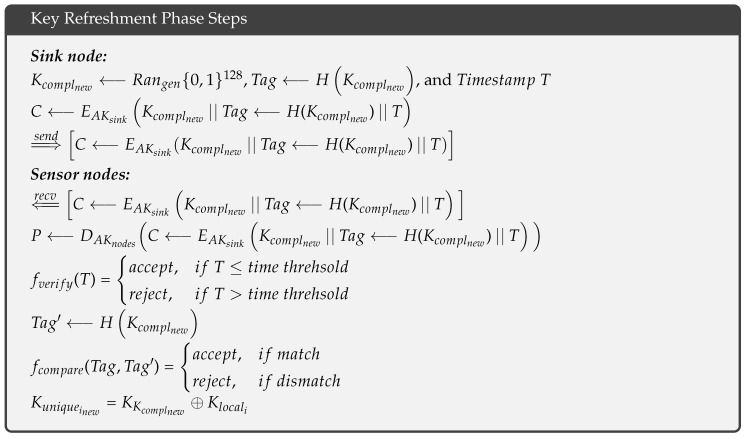


In the key refreshment phase, the sink node generates a new random complementary key $K_{compl_{new}}⟵Ran_{gen}{\left\{0,1\right\}}^{128}$, computes its hash value $Tag⟵H(K_{compl_{new}})$, and calculates a timestamp *T*. The sink node then encrypts these values using its asymmetric key $AK_{sink}$ and sends the cipher to the neighboring sensor nodes as follows: $\overset{send}{\Rightarrow }\left[C⟵E_{AK_{sink}}\left(K_{compl_{new}}\;||\;Tag⟵H\left(K_{compl_{new}}\right)\;\left|\right|\;T\right)\right]$. These neighbors forward the cipher to their neighbors in a multihop fashion until all of the sensor nodes have received the cipher $\overset{recv}{⇐}\left[C⟵E_{AK_{sink}}\left(K_{compl_{new}}\;||\;Tag⟵H\left(K_{compl_{new}}\right)\;\left|\right|\;T\right)\right]$. Because the sensor nodes are loaded with the asymmetric key $AK_{nodes}$ in the pre-deployment phase, a sensor node_i_ can decrypt the cipher as follows: $P⟵D_{AK_{nodes}}\left(C⟵E_{AK_{sink}}\left(K_{compl_{new}}\;||\;Tag⟵H\left(K_{compl_{new}}\right)\;\left|\right|\;T\right)\right)$ and verifies the timestamp $f_{verify}\left(T\right)$, based on a predefined threshold. If *T* exceeds the threshold, the sensor node_i_ rejects the cipher; otherwise, the node hashes the new complementary key ${Tag}^{\prime }⟵H\left(K_{compl_{new}}\right)$ and compares it with the received hash $f_{compare}\left(Tag,{Tag}^{\prime }\right)$ to ensure it has not received a modified new complementary key $K_{compl_{new}}$. If a mismatch is found, the sensor node_i_ rejects the cipher; otherwise, it produces its new unique key by XORing the new complementary key and its local key as follows: $K_{unique_{i_{new}}}=K_{compl_{new}}⊕K_{local_{i}}$.

## 4. Findings and Analyses

In this section, we analyze the efficiency and security of the proposed protocol in comparison to the schemes proposed in [26,35,36,37]. These analyses are based on the metrics presented in Table 1.

### 4.1. Efficiency Analysis

#### 4.1.1. Energy Consumption

Table 3 compares the energy consumption required by the proposed protocol and the corresponding schemes to perform a key distribution/establishment process between two nodes. (Because some schemes perform key distribution and others conduct key establishment, we refer to both terms as the “key distribution/establishment process.”). The experimental design and parameters are described in Appendix A.1.

The first part of the table includes eight rows to quantify the energy consumed by the nodes’ transceivers. The first row, “Th.N.F.Tx,” represents the theoretical number of frames sent by a node’s transmitter when performing a key distribution/establishment process without modeling wireless channel effects. The second row, “Av.N.F.Tx,” shows the average number of frames sent after modeling the wireless channel effects. The third row, “N.F.Rx,” shows the number of frames that a node’s receiver receives during the key distribution/establishment process. If a scheme involves exchanged frames for node discovery or clustering before the key distribution/establishment process, the fourth row, “N.F.ND.C,” represents the number of those frames. The fifth row, “T.Tx,” shows the time the node’s transmitter requires to send the frames. The sixth row, “T.Rx,” shows the time the node’s receiver requires to receive the frames. The seventh row, “E.TPO,” shows the energy consumed by the transmitter power output (TPO). The eighth row, “E.TRX,” shows the total energy consumed by the nodes’ transceivers.

The second part of the table quantifies the energy consumption of the nodes’ microcontrollers and includes eight rows. The first row, “S.C.K,” represents the search complexity for finding a common key, where *n* is the number of nodes, *P* is the key pool size, and $P_{C}$ is the probability that two nodes share a key. The next six rows show the time a node’s microcontroller requires to perform the various operations required to complete the key distribution/establishment process. The “T.MA.K” row shows the time a node’s microcontroller requires to find a common key; “T.MA.X” is the time a node’s microcontroller requires to perform an XOR operation; “T.MA.H” is the time a node’s microcontroller requires to perform a hashing operation; “T.MA.E” is the time a node’s microcontroller requires for encryption; and “T.MA.D” is the time a node’s microcontroller requires for decryption. When a scheme involves polynomial evaluation, “T.MA.P.E” is the time a node’s microcontroller requires for polynomial evaluation. The eighth row, “E.MA,” shows the total energy consumed by the nodes’ microcontrollers.

The last part of the table, “T.C.E,” shows the total energy consumption of the proposed protocol and the corresponding schemes.

As shown in Table 3, the nodes’ transceivers consume the lowest amount of energy in the proposed protocol, followed by scheme [37], at 2.75 mJ and 4.15 mJ, respectively; whereas, the nodes’ transceivers consume the largest amount of energy in scheme [35], followed by scheme [36] and scheme [26], at 295.77 mJ, 150.37 mJ, and 46.02 mJ, respectively.

In contrast, the nodes’ microcontrollers consume the lowest amount of energy in scheme [26], followed by scheme [36] and scheme [35], at 0.15 mJ, 1.29 mJ, and 27.50 mJ, respectively, and the largest amount of energy consumed is in the proposed protocol, followed by scheme [37], at 38.35 mJ and 38.23 mJ, respectively.

However, the total energy consumption in the proposed protocol is 41.10 mJ, which is the lowest relative to the total energy consumed by each of the corresponding schemes. This result is intuitive because the proposed protocol is designed to perform key distribution based on efficient data computation rather than data communication.

#### 4.1.2. Key Storage Overhead

**Figure 2:**
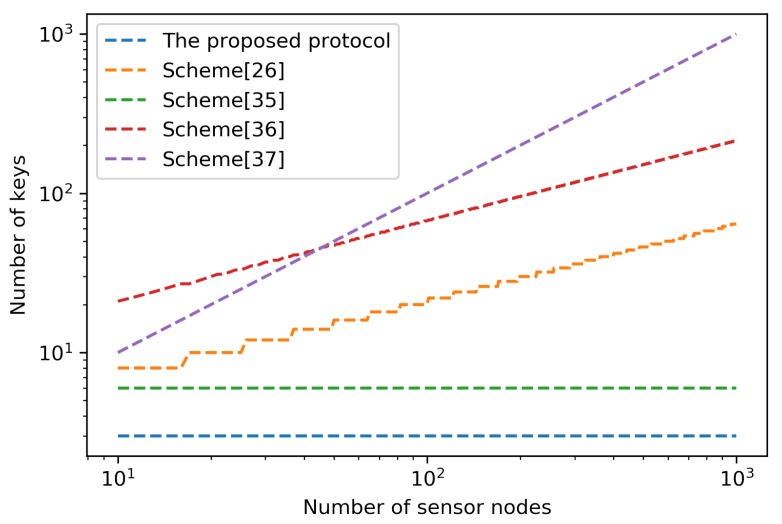
The magnitude of key storage overhead.

Table 4 illustrates the key storage overhead of the proposed protocol and the corresponding schemes. In the pre-deployment phase of the proposed protocol, each sensor node stores two keys, $AK_{nodes}$ and $K_{local}$. Then, in the key distribution phase, each sensor node prepares its unique key, $K_{unique}$. Therefore, the proposed protocol has the lowest key storage overhead for each sensor node compared to the corresponding schemes. The logarithmic graph presented in Figure 2 shows the magnitude of the key storage overhead as the number of sensor nodes increases. The graph clearly shows that the proposed protocol is advantageous because it requires the fewest keys compared to other schemes.

#### 4.1.3. Key Connectivity

**Figure 3:**
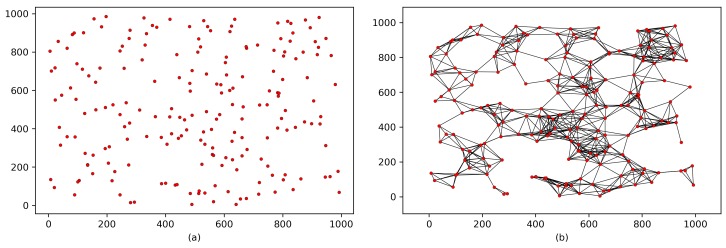
Modeling a WSN: (**a**) random deployment of sensor nodes; (**b**) wireless signal range of nodes’ transceivers.

To evaluate the key connectivity in each scheme, we model the entire WSN with a graph in which the vertices represent wireless sensor nodes and the edges represent links. Therefore, Figure 3a shows a random deployment of 200 nodes over an area of 1000 ft · 1000 ft. In Figure 3b, the black links indicate the wireless signal range of the nodes’ transceivers without applying either the proposed protocol or the corresponding schemes. The wireless signal range is based on the nodes’ transceiver modules (described in Appendix A.1).

**Figure 4:**
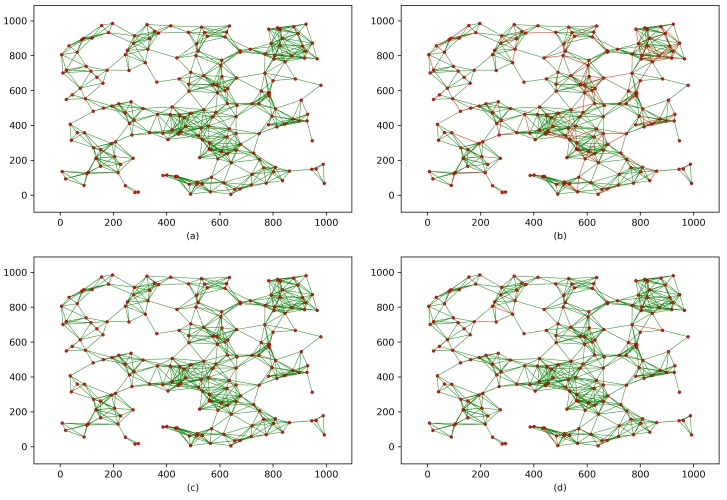

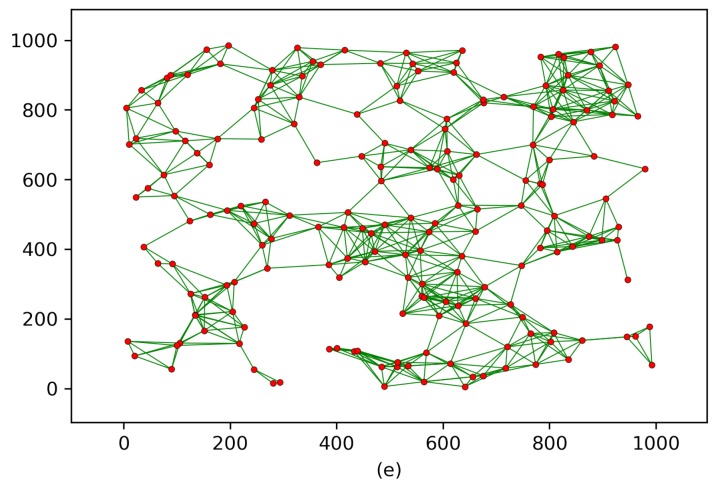
Key connectivity after implementing key distribution/establishment process for each scheme. (**a**) the proposed protocol; (**b**) Scheme [26]; (**c**) Scheme [35]; (**d**) Scheme [36] ; (**e**) scheme [37].

Figure 4 shows the implementation of the proposed protocol and the corresponding schemes on the WSN shown in Figure 3b. However, when two sensor nodes share a common key or key material within the same wireless range, the black links are converted to green links. In contrast, the red links indicate nodes that do not share a common key or any key materials. This modeling shows the key connectivity of each scheme, which can be defined as follows:

(5) $$The\;key\;connectivity\left(\%\right)=\frac{Secured\;links}{Total\;number\;of\;links}\times 100,$$

where the term “Securedlinks” includes any link between two nodes that share a common key or key materials, and “Totalnumberoflinks” counts all links in the WSN. Figure 4a,c,e show that the key connectivity is certain because nodes in these schemes are designed to have either a shared common key or key materials that lead to 100% key connectivity. In contrast, the key connectivities in Figure 4b,d reach only 87% and 99%, respectively.

### 4.2. Security Analysis

#### 4.2.1. Replay Attack

In a replay attack, an adversary captures a copy of exchanged frames to resend them later to the receiver for a deceptive purpose. The goal is for the receiver to believe that the resent messages are new messages; however, the receiver receives old information. This type of attack cannot be performed against the proposed protocol because a timestamp *T* is employed as a countermeasure in all three on-line phases: key distribution, post-key distribution and key refreshment. In the key distribution and key refreshment phases, the timestamp *T* is appended to each complementary key sent to the sensor nodes:

$\overset{send}{\Rightarrow }\left[C⟵E_{AK_{sink}}\left(K_{compl}\;||\;Tag⟵H\left(K_{compl}\right)\;\left|\right|\;T\right)\right]$

$\overset{send}{\Rightarrow }\left[C⟵E_{AK_{sink}}\left(K_{compl_{new}}\;||\;Tag⟵H\left(K_{compl_{new}}\right)\;\left|\right|\;T\right)\right]$.

In the post-key-distribution phase , each sensor node appends a timestamp *T* to each frame sent to the sink node.

$\overset{send}{\Rightarrow }\left[Id_{i}{}^{\prime }\;\left|\right|\;C⟵E_{K_{unique_{i}}}^{⊥}\left(D\;||\;Id_{i}\;\left|\right|\;T\right)\right]$.

This timestamp allows the sink node to validate whether the received data are replayed data. However, none of the corresponding schemes implement a countermeasure against replay attacks.

#### 4.2.2. Man-in-the-Middle Attack

During a man-in-the-middle attack, an adversary secretly intercepts frames from the sender and likely modifies them. Then, the adversary resends the frames to the receiver. This process occurs without the knowledge of the sender and receiver; therefore, both parties assume that they are communicating directly with one another. However, the proposed protocol is secure against man-in-the-middle attacks because in the pre-deployment phase, an asymmetric key pair is generated, renamed $AK_{sink}$ and $AK_{nodes}$, and loaded into the sink node, and the sensor nodes, respectively.

$\left\{K_{P},K_{R}\right\}⟵RSA_{gen}$

$K_{P}\overset{def}{=}AK_{sink}\;\;\mathrm{and}\;\;K_{R}\overset{def}{=}AK_{nodes}.$

Thus, during the key distribution and key refreshment phases, when the sink node encrypts the complementary key with $AK_{sink}$:

$C⟵E_{AK_{sink}}\left(K_{compl}\;||\;Tag⟵H\left(K_{compl}\right)\;\left|\right|\;T\right).$

Only the sensor nodes are able to decrypt the cipher because they already possess one key of the asymmetric key pair.

Additionally, in the post-key distribution phase, each sensor node encrypts data with its unique key that is only in the possession of the sensor node and the sink node.

$C⟵E_{K_{unique_{i}}}^{⊥}\left(D\;||\;Id_{i}\;\left|\right|\;T\right).$

Therefore, an adversary cannot impersonate either the sink node or any sensor node in the proposed protocol. Scheme [37] is vulnerable to man-in-the-middle attacks; however, the other compared schemes are not subjected to this type of attack.

#### 4.2.3. Node Capture Attack

**Figure 5:**
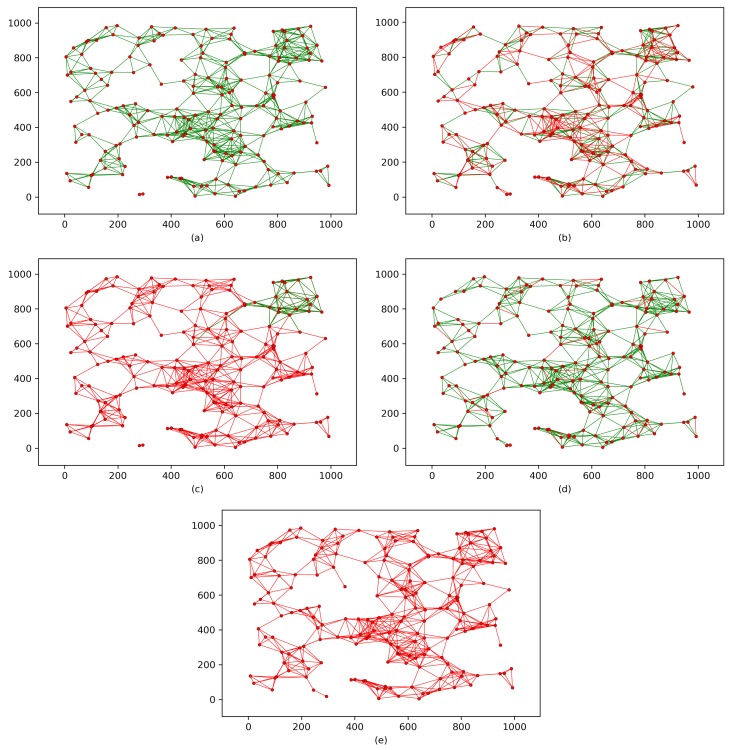
Schemes’ resilience against node capture attacks. (**a**) The proposed protocol; (**b**) Scheme [26]; (**c**) Scheme [35]; (**d**) Scheme [36] ; (**e**) Scheme [37].

To investigate the resilience of the proposed protocol and the corresponding schemes against node capture attacks, node capture attacks must be launched on the key connectivity of each scheme. Therefore, as shown in Figure 5, we mount node capture attacks on the key connectivity of each scheme presented in Figure 4. Thus, Figure 5 shows each scheme’s resilience against node capture attacks. The impact of a node capture attack can be defined as follows:

(6) $$The\;impact\;of\;a\;node\;capture\;attack\;on\;a\;WSN\left(\%\right)=\frac{Compromised\;links}{Total\;number\;of\;secured\;links}\times 100,$$

where “Compromisedlinks” indicates the number of links that are compromised after a random number of sensor nodes have been captured, and “Totalnumberofsecuredlinks” counts any link between two nodes that share a common key or key materials. However, each scheme has a different design; thus, for fairness, we assume the following:

1. An adversary is able to physically capture 5% of the sensor nodes randomly (i.e., 10 sensor nodes in the example network).
2. Because capturing the sink node will compromise any given WSN, in Assumption 1, node capture does not include the sink node.
3. Capturing a sensor node reveals all the data that node contains. For example, if the captured sensor node contains data that reveal information about other nodes’ common keys or keys materials, those keys are also compromised.

In Figure 5a, the key connectivity of the proposed protocol does not change after 10 sensor nodes are captured, which indicates that the proposed protocol is secure against node capture attacks. However, the key connectivities of the networks in Figure 5b,c are decreased by 51% and 75%, respectively (assuming the network in Figure 5c has 4 clusters and that some of the compromised nodes are located in 3 different clusters). The key connectivity in Figure 5d is only slightly affected, decreasing by only 6%. In contrast, the network in Figure 5e is heavily impacted, and the key connectivity is decreased by 100%.

## 5. Formal Verification

To formally prove the security and soundness of the proposed protocol, we utilize ProVerif, an automatic cryptographic protocol verifier. ProVerif is a powerful tool for automatically analyzing the security of cryptographic protocols and verifying them in a formal model.

In this section, we present the verification results pertaining to reachability and secrecy, correspondence assertions (authentication), and observational equivalences.

### 5.1. Reachability and Secrecy

**Figure 6:**
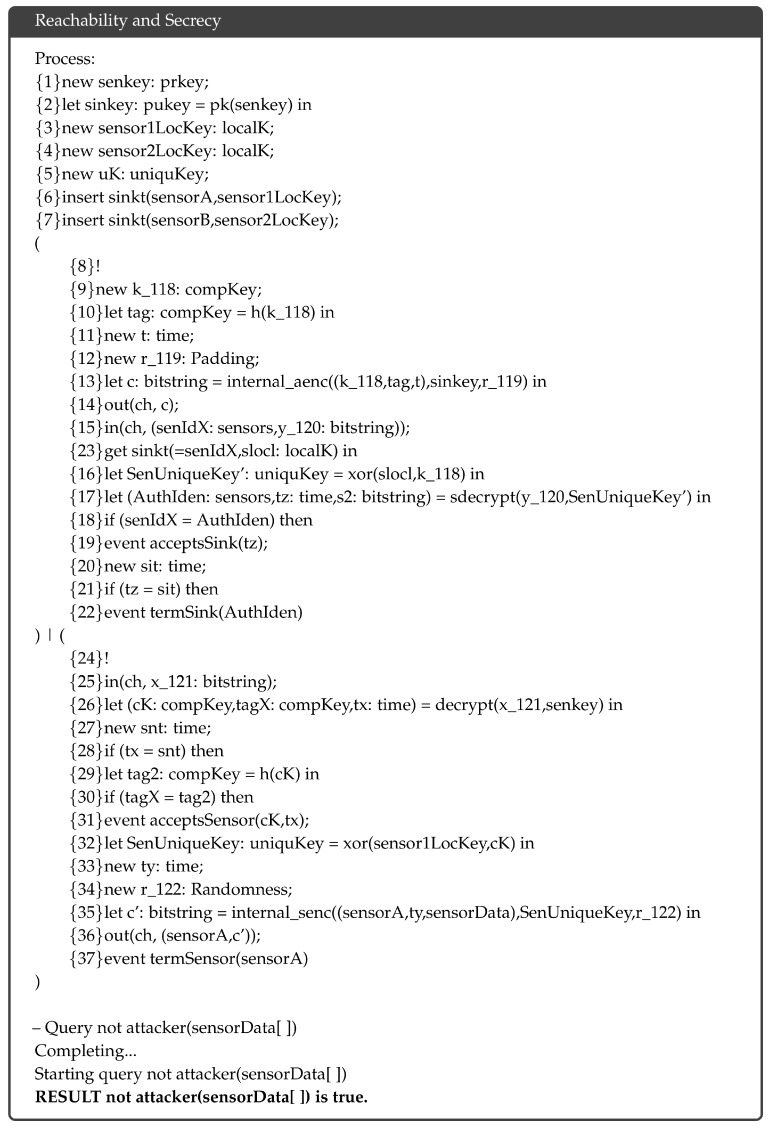
Verification result of reachability and secrecy.

ProVerif provides proof of reachability and secrecy properties by investigating the reachability of a term *x* by an adversary A. Based on the results, the secrecy of *x* can be assessed with respect to the modeled protocol. In the proposed protocol, we test whether sensor data “sensorData” are available to A. Figure 6 shows the complete verification result. The result concludes, “RESULT not attacker(sensorData[ ]) is true”, meaning that “sensorData” is unreachable, and an attack cannot be conducted against the protocol successfully.

### 5.2. Correspondence Assertions

**Figure 7:**
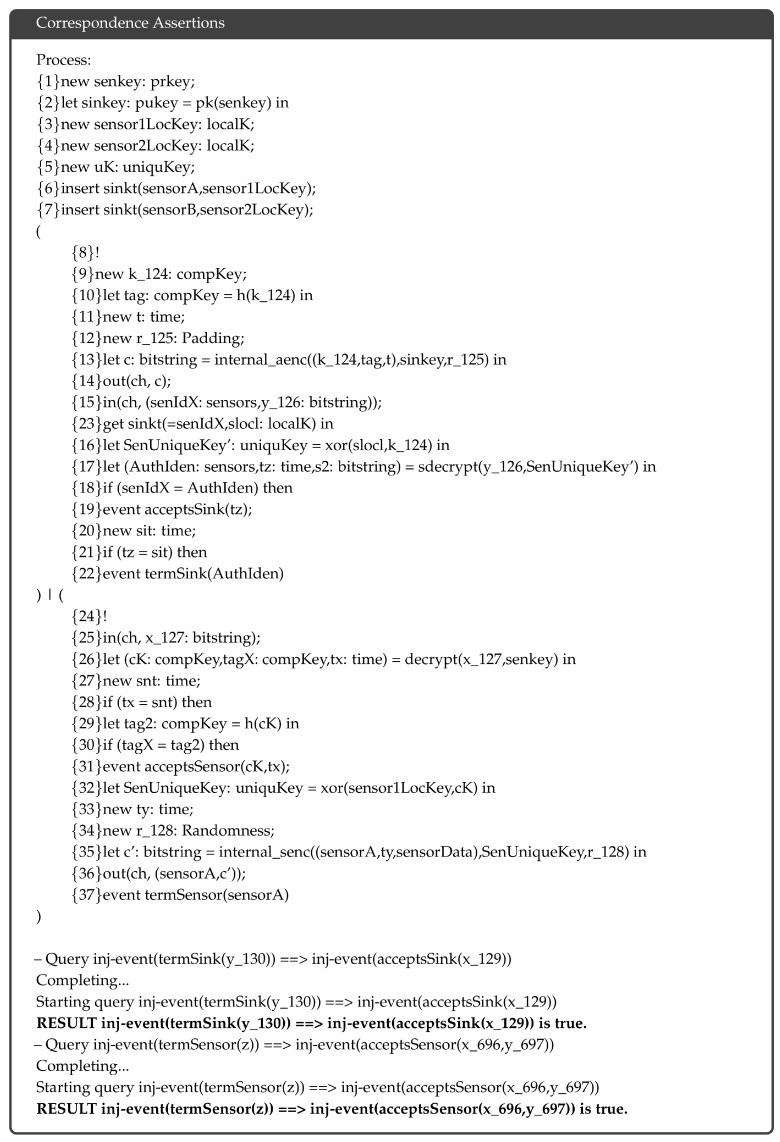
Verification result of authentication.

In ProVerif, authentication can be modeled using a sequence of events defined as correspondence assertions. We apply a sequence of events to verify the authentication of the sink node and the complementary key to the sensor nodes and the authentication of the sensor nodes and encrypted data to the sink node. Figure 7 shows the complete verification result. The verification confirmes that the proposed protocol achieves successful authentication.

### 5.3. Observational Equivalence

**Figure 8:**
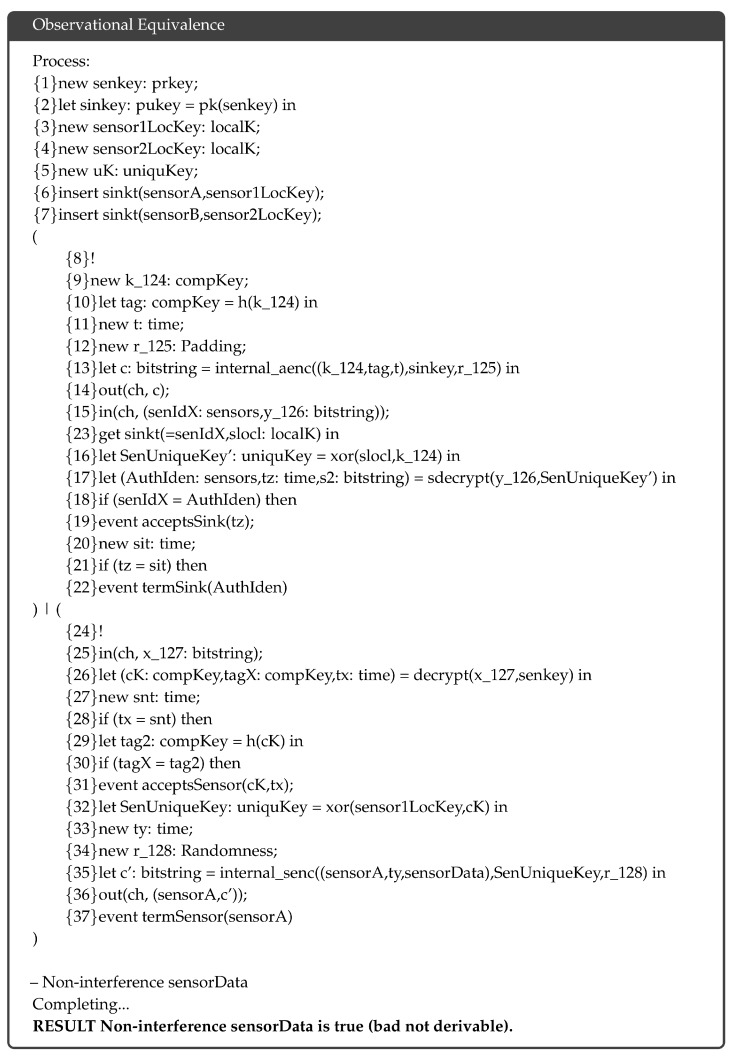
Verification result of observational equivalence.

In applied π-calculus terminology, two processes $p_{1}$ and $p_{2}$ have observational equivalence $\left(p_{1}\approx p_{2}\right)$ when they are indistinguishable through observation. ProVerif can prove observational equivalence such as strong secrecy, in which an adversary 𝒜 cannot distinguish when a cipher changes. We leverage this feature to prove that the proposed protocol is semantically secure and that the 𝒜 cannot learn anything from the cipher. Figure 8 shows the complete verification result. The analysis showes that the sensor data “sensorData” in the proposed protocol are observationally equivalent and that the 𝒜 cannot distinguish when they change because the data are encrypted by a probabilistic algorithm, as described in the post-key distribution phase.

## 6. Conclusions

In this work, we propose a practical key distribution protocol that can be implemented above the IEEE 802.15.4 standard to secure the wireless communication of resource-constrained sensor nodes. We utilized existing cryptographic primitives to design a protocol that maintains a tradeoff between efficiency and security. We conducted simulation, hardware implementations, and modeling to compare the proposed protocol to the existing solutions. Moreover, we conducted formal verifications to prove the soundness and the security of our proposed protocol. The proposed protocol provides low energy and memory consumption, certain key connectivity, and security against: replay attack, man-in-the-middle attack, and node capture attack. The overall results show that the proposed protocol is more efficient and secure than the corresponding schemes.

Future work includes examining more advanced methods to enhance the energy consumption of the proposed protocol. For example, using more efficient algorithms to perform data computation. Furthermore, investigating additional types of attacks against the proposed protocol to increase its security.

## Figures and Tables

**Figure A1:**
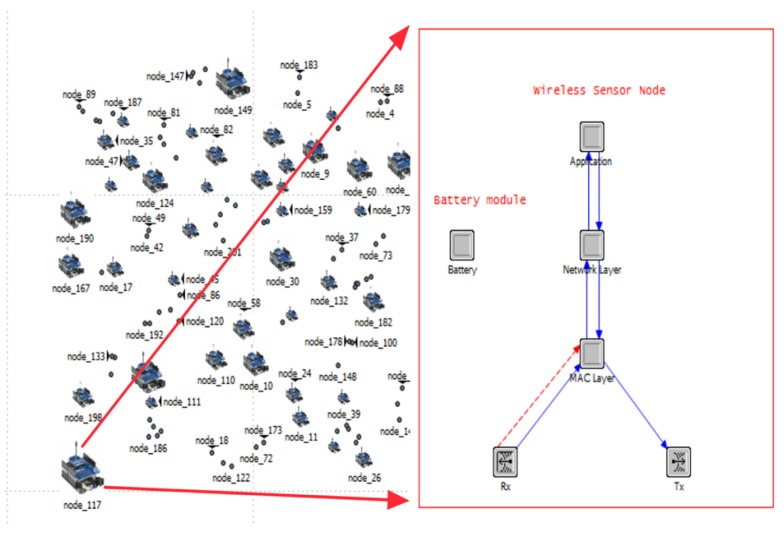
Our wireless sensor node model.

**Table A1:** Experiment Parameters.

Description	Parameters	Values
**Channel**	Data Rate	250 kbps
	Frame Size	1024 bits
	Transmission power	0 dBm
	Modulation	bpsk
	Receiver Sensitivity	−92 dBm
**Transceiver**	Tx Current draw	45 mA @ 3.3 VDC
	Rx Current draw	50 mA @ 3.3 VDC
**Microcontroller**	Microcontroller	3.2 mA @ 4.5 V
**Power Requirements**	Tx power consumption	148.5 mW
	Rx power consumption	165 mW
	Microcontroller power consumption	14.4 mW

**Table 1:** Evaluation Metrics.

	Metric	Definition
Efficiency	Energy consumption	The amount of energy consumed during the key distribution/key establishment process.
	Storage overhead	The memory required to store keys or keys materials.
	Key connectivity	The percentage of available links in a WSN, calculated as the number of secured links divided by the total links.
Security	Replay attack	The ability of an adversary to replay any of the corresponding frames.
	Man-in-the-middle attack	The ability of an adversary to impersonate any sensor node or sink node.
	Resiliency to node capture attack	The impact percentage of a node capture attack on WSN key connectivity, calculated as the number of compromised links over the number of secured links.

**Table 2:** Notation for the Proposed Protocol.

Notation	Description
y⟵x	*y* is generated by *x*.
$x\overset{def}{=}y$	*x* is defined as *y*.
x:=y	*y* is assigned to *x*.
H()	One-way hash function.
||	Concatenation.
$\overset{send}{\Rightarrow }\left[x\right]$	Sending message *x*.
$\overset{recv}{⇐}\left[x\right]$	Receiving message *x*.
$E_{k}\left(y\right)$	*y* is encrypted with *k*.
$E_{k}^{⊥}\left(y\right)$	*y* is encrypted with *k* using algorithm ⊥.
f()	Function to compare or verify.
P()	Probability function.
F:A↦B	Function maps *A* to *B*.
*P*	Plaintext.
*C*	Cipher text.
Id	Node identification.
*T*	Timestamp.
*D*	Data.

**Table 3:** Energy consumption of each key distribution scheme.

Descriptions		Schemes	Our Protocol	Scheme [26]	Scheme [35]	Scheme [36]	Scheme [37]
	Parameters						
The parameters that contribute to the energy consumption of nodes’ transceivers.	**Th.N.F.Tx**		1	27	6	95	2
	**Av.N.F.Tx**		3	39	13	120	4
	**N.F.Rx**		1	27	6	95	2
	**N.F.ND.C**		NA	2	201	NA	NA
	**T.Tx**		12.29 ms	159.74 ms	876.54 ms	491.52 ms	16.38 ms
	**T.Rx**		4.10 ms	110.59 ms	847.87 ms	389.12 ms	8.19 ms
	**E.TPO**		0.01 mJ	0.16 mJ	0.88 mJ	0.49 mJ	0.02 mJ
	**E.TRX**		2.75 mJ	46.02 mJ	295.77 mJ	150.37 mJ	4.15 mJ
The parameters that contribute to the energy consumption of nodes’ microcontroller.	**C.S.K**		𝒪(1)	$O(2\cdot \sqrt{n-1})$	𝒪(1)	$O(\sqrt{-P\cdot log(1-P_{c})}){\;}^{1}$	𝒪(*n*)
	**T.MA.K**		NA	10.08 ms	NA	89.31 ms	170.02 ms
	**T.MA.X**		0.35 ms	NA	NA	NA	NA
	**T.MA.H**		177.82 ms	NA	NA	NA	NA
	**T.MA.E**		982 ms	*t* ${}^{2}$	NA	NA	982 ms
	**T.MA.D**		1502.90 ms	*t* ${}^{2}$	NA	NA	1502.90 ms
	**T.MA.P.E**		NA	NA	1909.83 ms	NA	NA
	**E.MA**		38.35 mJ	0.15 mJ	27.50 mJ	1.29 mJ	38.23 mJ
Total energy consumption	**T.E.C**		41.10 mJ	46.17 + 2$t^{2}$ mJ	323.28 mJ	151.65 mJ	42.39 mJ

${}^{1}$ The key pool size *P* is equal to the number of sensor nodes multiplied by 10, and $P_{c}$ is equal to 0.99%. ${}^{2}$ Here, *t* denotes an unknown time; the time cannot be determined because the scheme involves encryption and decryption during the key distribution process, and the source does not specify the type of encryption and decryption algorithm used.

**Table 4:** Key Storage overhead for each scheme.

Scheme	Key Storage Overhead
The proposed protocol	3
Scheme [26]	$2\cdot \sqrt{n-1}$
Scheme [35]	6
Scheme [36]	$\sqrt{-P\cdot log(1-P_{c})}{\;}^{1}$
Scheme [37]	*n*

*n* represents the number of nodes. ${}^{1}$ The key pool size *p* is equal to the number of sensor nodes multiplied by 10, and $P_{c}$ is equal to 0.99%.

## Appendix A. Methodology

### Appendix A.1. Experiment Design and Parameters

The experimental design includes two parts: simulation and hardware implementation. In the first part, we utilize the OPNET Modeler to design a model for a wireless sensor node and then used this model to conduct simulations for a network of 200 wireless sensor nodes, as shown in Figure A1. Our sensor node model calculates the energy consumption of a node’s transceiver, including both the TPO and the energy consumption caused by wireless channel effects, based on the models described in Appendix A.2 and Appendix A.3, respectively.

In the second part, because the energy consumption by a node’s microcontroller cannot be simulated, we implement the proposed protocol and the compared schemes on a real microcontroller and measure the time these schemes require to perform key distribution/establishment processes. Then, we calculate the energy consumption of the node’s microcontroller based on the model described in Appendix A.2.

Moreover, in this experiment, the transceiver parameters of our sensor node model are based on XBee transceiver S1 [41], and the microcontroller used in the implementation is an Atmega328p [42] (clocked at 16 MHz). The following table shows these parameters.

### Appendix A.2. Energy Consumption of Wireless Sensor Node

The energy consumption of a wireless sensor node is the total amount of energy consumed by the node’s transceiver, microcontroller, and sensors, as indicated in Equation (A1). Because our research considers key distribution security and efficiency regardless of the network’s application, we ignore the energy consumed by the sensors because that consumption is based on sensor applications regardless of the underlying key distribution/establishment algorithm. Thus, in this paper, the energy consumption of a wireless sensor node is calculated by Equation (A2):

(A1) $$E_{sensor\;node}=E_{transceiver}+E_{microcontroller}+E_{sensor}$$

(A2) $$E_{sensor\;node}=E_{transceiver}+E_{microcontroller}.$$

The energy consumption is generally calculated by Equation (A3):

(A3) Energy(J)=Power(W)·Time(S),

where Power(W) is calculated by Equation (A4):

(A4) Power(W)=Voltage(V)·Current(A).

Based on Equation (A3), we calculate the energy consumption of nodes’ transceivers and microcontrollers. The following subsections describe these calculations.

#### Appendix A.2.1. Energy Consumption of the Transceiver

The energy consumption of a node’s transceiver $E_{transceiver}$, given in Equation (A2), is the energy consumed by the node’s transmitter $E_{Tx}$ and its receiver $E_{Rx}$. However, the energy consumption of the node’s transmitter $E_{Tx}$ is the total energy consumed by the TPO $E_{Tx_{TPO}}$ and the transmitter electronics $E_{Tx_{elec}}$, which can be found by the following equation:

(A5) $$E_{Tx}=E_{Tx_{TPO}}+E_{Tx_{elec}},$$

where the energy consumed by $E_{Tx_{TPO}}$ is found by Equation (A6):

(A6) $$E_{Tx_{TPO}}=Tx_{power_{output}}\cdot Tx_{time},$$

where $Tx_{power_{output}}$ is the TPO, and it depends on the transceiver module (described in Appendix A.1). $Tx_{time}$ is the time that a node’s transmitter takes to send one frame and is found by Equation (A7):

(A7) $$Tx_{time}=\left(\frac{Fr_{Size}}{Tx_{DR}}\right),$$

where $Fr_{Size}$ is the frame size, and $Tx_{DR}$ is the data rate of the transmitter. The transmitter data rate depends on the transceiver module (described in Appendix A.1).

The energy consumed by the transmitter electronics $E_{Tx_{elec}}$ can be calculated as follows:

(A8) $$E_{Tx_{elec}}=Tx_{time}\cdot \left(\left(Tx_{elec_{current}}\cdot Tx_{elec_{voltage}}\right)+\left(MA_{Tx_{current}}\cdot MA_{Tx_{voltage}}\right)\right),$$

where $Tx_{time}$ is found as shown in Equation (A7). $Tx_{elec_{current}}$ and $Tx_{elec_{voltage}}$ are the respective current and voltage required by the transmitter’s electronics, and $MA_{Tx_{current}}$ and $MA_{Tx_{voltage}}$ are the respective current and voltage required by a node’s microcontroller to run the transmitter.

The energy consumption of the node’s receiver $E_{Rx}$ can be calculated as follows:

(A9) $$E_{Rx}=Rx_{time}\cdot \left(\left(Rx_{current}\cdot Rx_{voltage}\right)+\left(MA_{Rx_{current}}\cdot MA_{Rx_{voltage}}\right)\right),$$

where $Rx_{time}$ is the time that a node’s receiver requires to receive one frame, which can be found by Equation (A10). $Rx_{current}$ and $Rx_{voltage}$ are the respective current and voltage required by a node’s receiver, and $MA_{Rx_{current}}$ and $MA_{Rx_{voltage}}$ are the respective current and voltage required by a node’s microcontroller to run the receiver.

(A10) $$Rx_{time}=\left(\frac{Fr_{Size}}{Rx_{DR}}\right),$$

where $Fr_{Size}$ is the frame size, and $Rx_{DR}$ is the data rate of the receiver. The receiver’s data rate depends on the transceiver module (described in Appendix A.1).

#### Appendix A.2.2. Energy Consumption of the Microcontroller

The energy consumption of a node’s microcontroller, $E_{microcontroller}$, presented in Equation (A2), represents the energy consumed by a node’s microcontroller, $E_{MA}$, and can be found using the following equation:

(A11) $$E_{MA}=MA_{Time_{operate}}\cdot MA_{current}\cdot MA_{voltage},$$

$MA_{Time_{operate}}$ is the time required by the microcontroller to execute the core algorithm of a key distribution/establishment process. $MA_{current}$ and $MA_{voltage}$ are the respective current and voltage required by the microcontroller.

### Appendix A.3. Modeling Wireless Channel Effects

When our sensor node model receives a frame, it calculates the received power $P_{Rx}$ of the frame as follows:

(A12) $$P_{Rx}=P_{Tx}\cdot G_{Tx}\cdot G_{Rx}\cdot P_{Loss},$$

where $P_{Tx}$ is the transmitter power, $G_{Tx}$ is the transmitter antenna gain, $G_{Rx}$ is the receiver antenna gain, and $P_{Loss}$ is the path loss in free space, which can be found by the following equation:

(A13) $$P_{Loss}={\left(\frac{\lambda }{4\pi D}\right)}^{2},$$

where λ is the wavelength, and *D* is the distance.

If the received power is less than the receiver sensitivity, our model discards the frame because the receiver is unable to decode it. However, if the received power is greater than or equal to the receiver’s sensitivity, our model calculates the noise in each frame caused by interference from other frames and then calculates the signal-to-noise ratio (SNR) based on Equation (A14):

(A14) $$SNR=10\log_{10}\left(\frac{P_{Rx}}{N_{Intern}}\right).$$

Then, our model calculates the bit error rate (BER) in the received frame, which is calculated based on the chosen modulation curve and the SNR. Finally, it calculates the percentage of errors in the received frame by the following equation:

(A15) $$F_{Errors}=\left(\frac{BER}{F_{size}}\right),$$

where $F_{size}$ is the frame size. If the percentage of errors in the received frame $F_{Errors}$ exceeds a specified threshold, the model discards the frame.

### Appendix A.4. Fast Modular Exponentiation Algorithm

The proposed protocol relies on modular exponentiation in the key distribution phase. The running time for an ordinary modular exponentiation algorithm is exponential with the magnitude of the exponent *e* (linear complexity); thus the algorithm is not a practical. To make the proposed protocol practical in resource-constrained nodes, we utilized the square-and-multiply algorithm, Algorithm A1, which has a polynomial running time in the length of *e* (logarithmic complexity).

**Algorithm A1** Square-and-Multiply

## References

[1] Wan L., Han G., Shu L., Feng N., Zhu C., Lloret J. (2015). Distributed parameter estimation for mobile wireless sensor network based on cloud computing in battlefield surveillance system. IEEE Access.

[2] Trasviña-Moreno C.A., Blasco R., Marco Á., Casas R., Trasviña-Castro A. (2017). Unmanned aerial vehicle based wireless sensor network for marine-coastal environment monitoring. Sensors.

[3] Noel A.B., Abdaoui A., Elfouly T., Ahmed M.H., Badawy A., Shehata M.S. (2017). Structural health monitoring using wireless sensor networks: A comprehensive survey. IEEE Commun. Surv. Tutor..

[4] Lin J.R., Talty T., Tonguz O.K. (2015). A blind zone alert system based on intra-vehicular wireless sensor networks. IEEE Trans. Ind. Inf..

[5] Liang T., Yuan Y.J. (2016). Wearable medical monitoring systems based on wireless networks: A review. IEEE Sens. J..

[6] Iqbal Z., Kim K., Lee H.N. (2017). A Cooperative Wireless Sensor Network for Indoor Industrial Monitoring. IEEE Trans. Ind. Inf..

[7] Bapat V., Kale P., Shinde V., Deshpande N., Shaligram A. (2017). WSN application for crop protection to divert animal intrusions in the agricultural land. Comput. Electron. Agric..

[8] Aponte-Luis J., Gómez-Galán J.A., Gómez-Bravo F., Sánchez-Raya M., Alcina-Espigado J., Teixido-Rovira P.M. (2018). An Efficient Wireless Sensor Network for Industrial Monitoring and Control. Sensors.

[9] Antolín D., Medrano N., Calvo B., Pérez F. (2017). A wearable wireless sensor network for indoor smart environment monitoring in safety applications. Sensors.

[10] Adu-Manu K.S., Tapparello C., Heinzelman W., Katsriku F.A., Abdulai J.D. (2017). Water Quality Monitoring Using Wireless Sensor Networks: Current Trends and Future Research Directions. ACM Trans. Sensor Netw..

[11] Aguirre E., Lopez-Iturri P., Azpilicueta L., Redondo A., Astrain J.J., Villadangos J., Bahillo A., Perallos A., Falcone F. (2017). Design and Implementation of Context Aware Applications with Wireless Sensor Network Support in Urban Train Transportation Environments. IEEE Sens. J..

[12] Mainetti L., Patrono L., Vilei A. Evolution of wireless sensor networks towards the internet of things: A survey. Proceedings of the 19th International Conference on Software, Telecommunications and Computer Networks, SoftCOM 2011.

[13] Lazarescu M.T. (2013). Design of a WSN platform for long-term environmental monitoring for IoT applications. IEEE J. Emerg. Sel. Top. Circuits Syst..

[14] Kocakulak M., Butun I. An overview of Wireless Sensor Networks towards internet of things. Proceedings of the 2017 IEEE 7th Annual Computing and Communication Workshop and Conference (CCWC).

[15] Flammini A., Sisinni E. (2014). Wireless sensor networking in the internet of things and cloud computing era. Procedia Eng..

[16] IEEE 802 Working Group (2011). IEEE Standard for Local and Metropolitan Area Networks—Part 15.4: Low-Rate Wireless Personal Area Networks (LR-WPANs).

[17] Alshammari M.R., Elleithy K.M. Efficient key distribution protocol for wireless sensor networks. Proceedings of the 2018 IEEE 8th Annual Computing and Communication Workshop and Conference (CCWC).

[18] Akyildiz I.F., Su W., Sankarasubramaniam Y., Cayirci E. (2002). Wireless sensor networks: A survey. Comput. Netw..

[19] Zhang J., Varadharajan V. (2010). Wireless sensor network key management survey and taxonomy. J. Netw. Comput. Appl..

[20] Shim K.A. (2016). A survey of public-key cryptographic primitives in wireless sensor networks. IEEE Commun. Surv. Tutor..

[21] Mahajan P., Sardana A. (2012). Key distribution schemes in wireless sensor networks: Novel classification and analysis. Advances in Computing and Information Technology.

[22] Bala S., Sharma G., Verma A.K. A survey and taxonomy of symmetric key management schemes for wireless sensor networks. Proceedings of the CUBE International Information Technology Conference.

[23] Liao Y.H., Lei C.L., Wang A.N. A Robust Grid-Based Key Predistribution Scheme for Sensor Networks. Proceedings of the 2009 Fourth International Conference on Innovative Computing, Information and Control (ICICIC).

[24] Quy N.X., Kumar V. A high connectivity pre-distribution key management scheme in grid-based wireless sensor networks. Proceedings of the 2008 International Conference on Convergence and Hybrid Information Technology.

[25] Wang N.C., Chen Y.L., Chen H.L. (2014). An Efficient Grid-Based Pairwise Key Predistribution Scheme for Wireless Sensor Networks. Wirel. Pers. Commun..

[26] Chan H., Perrig A. PIKE: Peer intermediaries for key establishment in sensor networks. Proceedings of the INFOCOM 2005—24th Annual Joint Conference of the IEEE Computer and Communications Societies.

[27] Schneier B. (2007). Applied Cryptography: Protocols, Algorithms, and Source Code In C.

[28] Shamir A. (1979). How to share a secret. Commun. ACM.

[29] Hu T., Chen D., Tian X. An enhanced polynomial-based key establishment scheme for wireless sensor networks. Proceedings of the 2008 International Workshop on Education Technology and Training & 2008 International Workshop on Geoscience and Remote Sensing.

[30] Li X., Shen J. A novel key pre-distribution scheme using one-way hash chain and bivariate polynomial for wireless sensor networks. Proceedings of the 2009 3rd International Conference on Anti-counterfeiting, Security, and Identification in Communication.

[31] Ito H., Miyaji A., Omote K. RPoK: A strongly resilient polynomial-based random key pre-distribution scheme for multiphase wireless sensor networks. Proceedings of the 8th Grobal Communications Conference Exhibition & Industry Forum, IEEE GLOBECOM 2010, Institute of Electrical and Electronics Engineers (IEEE).

[32] Dai H., Xu H. An improved polynomial-based key predistribution scheme for wireless sensor networks. Proceedings of the 2010 IEEE Global Telecommunications Conference GLOBECOM 2010.

[33] Delgosha F., Ayday E., Fekri F. MKPS: A multivariate polynomial scheme for symmetric key-establishment in distributed sensor networks. Proceedings of the 2007 International Conference on Wireless Communications and Mobile Computing.

[34] Das A.K., Sengupta I. An effective group-based key establishment scheme for large-scale wireless sensor networks using bivariate polynomials. Proceedings of the 2008 3rd International Conference on Communication Systems Software and Middleware and Workshops (COMSWARE ’08).

[35] Baburaj E. (2017). Polynomial and multivariate mapping-based triple-key approach for secure key distribution in wireless sensor networks. Comput. Electr. Eng..

[36] Eschenauer L., Gligor V.D. A key-management scheme for distributed sensor networks. Proceedings of the 9th ACM Conference on Computer and Communications Security.

[37] Yu Z. The scheme of public key infrastructure for improving wireless sensor networks security. Proceedings of the 2012 IEEE 3rd International Conference on Software Engineering and Service Science (ICSESS).

[38] Chung A., Roedig U. Efficient Key Establishment for Wireless Sensor Networks Using Elliptic Curve Diffie-Hellman. Proceedings of the 2nd European Conference on Smart Sensing and Context (EUROSSC2007).

[39] Nagy N., Nagy M., Akl S.G. (2008). Quantum Wireless Sensor Networks.

[40] Li J.S., Yang C.F. Quantum communication in distributed wireless sensor networks. Proceedings of the 2009 IEEE 6th International Conference on Mobile Adhoc and Sensor Systems.

[41] Sheet X.P.D. XBee-Datasheet.pdf. www.sparkfun.com/datasheets/Wireless/Zigbee.

[42] Atmel Atmel ATmega328P Datasheet. http://ww1.microchip.com/downloads/en/DeviceDoc/Atmel-8271-8-bit-AVR-Microcontroller-ATmega48A-48PA-88A-88PA-168A-168PA-328-328P_datasheet_Summary.pdf.

